# The Focused Assessment with Sonography in Cancer (FASC) Examination

**DOI:** 10.24908/pocus.v5i2.14428

**Published:** 2020-11-18

**Authors:** Peter C Nauka, Benjamin T Galen

**Affiliations:** 1 Albert Einstein College of Medicine and Montefiore Medical Center, Department of Internal Medicine, Residency Training Program Bronx, NY USA; 2 Albert Einstein College of Medicine and Montefiore Medical Center, Department of Internal Medicine, Division of Hospital Medicine Bronx, NY USA

**Keywords:** POCUS, FASC

## Abstract

Malignant effusions occur frequently in patients with cancer and are important to diagnose and treat. In this report, we describe a novel point-of-care ultrasound (POCUS) protocol to rapidly identify pleural effusion, pericardial effusion, and ascites: The Focused Assessment with Sonography in Cancer (FASC). This protocol utilizes six standard sonographic positions to identify the presence of fluid in common anatomic spaces. The FASC examination is intended for widespread use by oncologists and other clinicians who treat patients with cancer.

## Introduction

**Figure 1:**
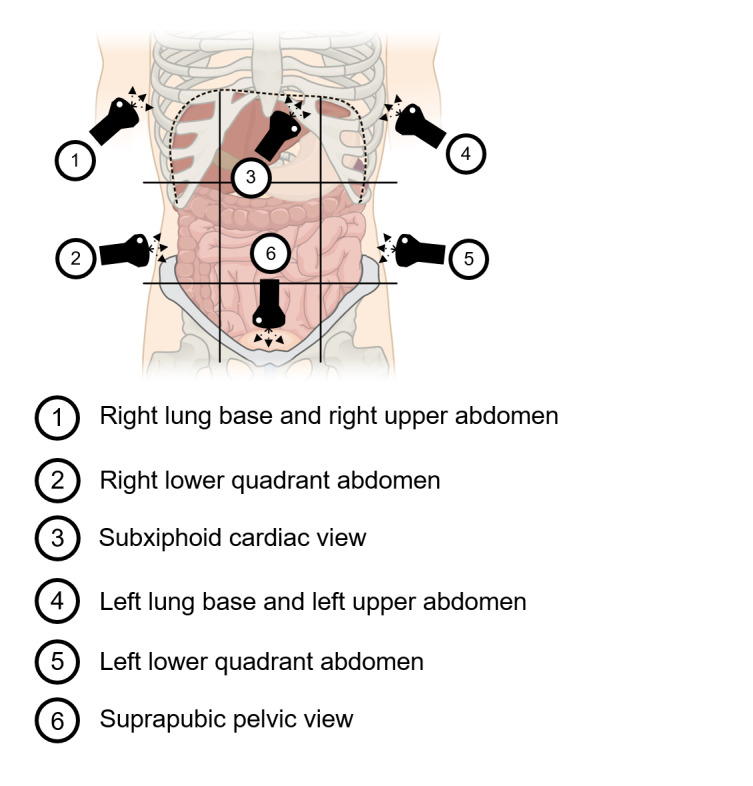
Protocol for Focused Assessment with Sonography in Cancer (FASC) Examination. Progression of scanning follows numbering from 1 to 6.

Clinicians in oncology, emergency medicine, hospital medicine, and primary care frequently encounter patients with cancer who develop fluid accumulation in the pleural, pericardial, abdominal, and pelvic spaces. Both solid tumor metastases and hematologic malignancies have the potential to cause third spacing of intravascular fluids via seeding to and disruption of serosal membranes and normal endothelium 1, 2. We propose a Focused Assessment with Sonography in Cancer (FASC) examination using point-of-care ultrasound (POCUS) to enable all clinicians to routinely and rapidly assess patients for pleural effusion, pericardial effusion, and ascites. Similar to other POCUS protocols like the Focused Assessment with Sonography in Trauma (FAST) examination, the FASC examination uses six views to detect fluid (Figure 1) 3, 4.

Third-space fluid accumulation leads to a range of symptoms for patients with cancer that can significantly reduce quality of life 3, 4, 5. Life-threatening complications, such as cardiac tamponade from a rapidly accumulating pericardial effusion, are important to detect expediently. Monitoring for recurrence of fluid accumulation in the pleural, pericardial, abdominal, and pelvic spaces is important in longitudinal outpatient care. Palliative procedures, such as intermittent drainage and indwelling catheters can decrease symptom burden from malignant exudates. The potential benefit for FASC examination is to diagnose and track fluid accumulation easily at the bedside or in the office, using POCUS as an adjunct to physical examination. 

POCUS is a portable, low-cost, and increasingly popular imaging modality in widespread use by many clinicians, but not yet by oncologists and hematologists 6. The use of POCUS in identifying fluid accumulation in potential spaces has been very well-established 7. This modality is well tolerated by patients and has excellent sensitivity and specificity compared to computerized tomography imaging (Table 1) 8, 9, 10. 

**Table 1:** Test characteristics of Point-of-Care Ultrasound (POCUS) in finding fluid. The diagnosis of pleural effusion and ascites were compared to computerized tomography imaging. Sensitivity and specificity for pericardial effusion were determined by comparing image interpretation by non-cardiologists to echocardiography boarded cardiologists (9, 10).

	Point-of-Care Ultrasound		
		Sensitivity	Specificity
Pleural Effusion	Visualization of effusion	93%	96%
Ascites	Visualization of fluid	96%	82%
Pericardial Effusion	Visualization of fluid	96%	98%

## FASC Examination

### Setup and Patient Positioning

The goal of the FASC examination should be explained to the patient and family. Reassurance should be offered that the FASC examination is not painful and does not use ionizing radiation. For the FASC examination, the patient should be placed in the supine or semi-recumbent position. The patient should place both hands comfortably behind the head; this can improve visualization through the intercostal spaces 11. Flexion of the hips and knees will relax abdominal muscles for optimal windows 11. 

Depending on availability, either a phased array probe (typically 2 Mhz – 7.4 Mhz) or curvilinear transducer probe (2 - 5 Mhz) can be used for the entire FASC examination. Users should be familiar with basic adjustments on their machine, such as gain and depth. In conventional probe orientation for POCUS, the probe indicator is placed towards the patient’s right or towards the head, except for the subxiphoid cardiac view, which uses cardiology orientation (indicator to patient’s left). 

Position 1 and 4. Detecting Pleural Effusions and Ascites in the Upper Abdominal Quadrants

**Figure 2:**
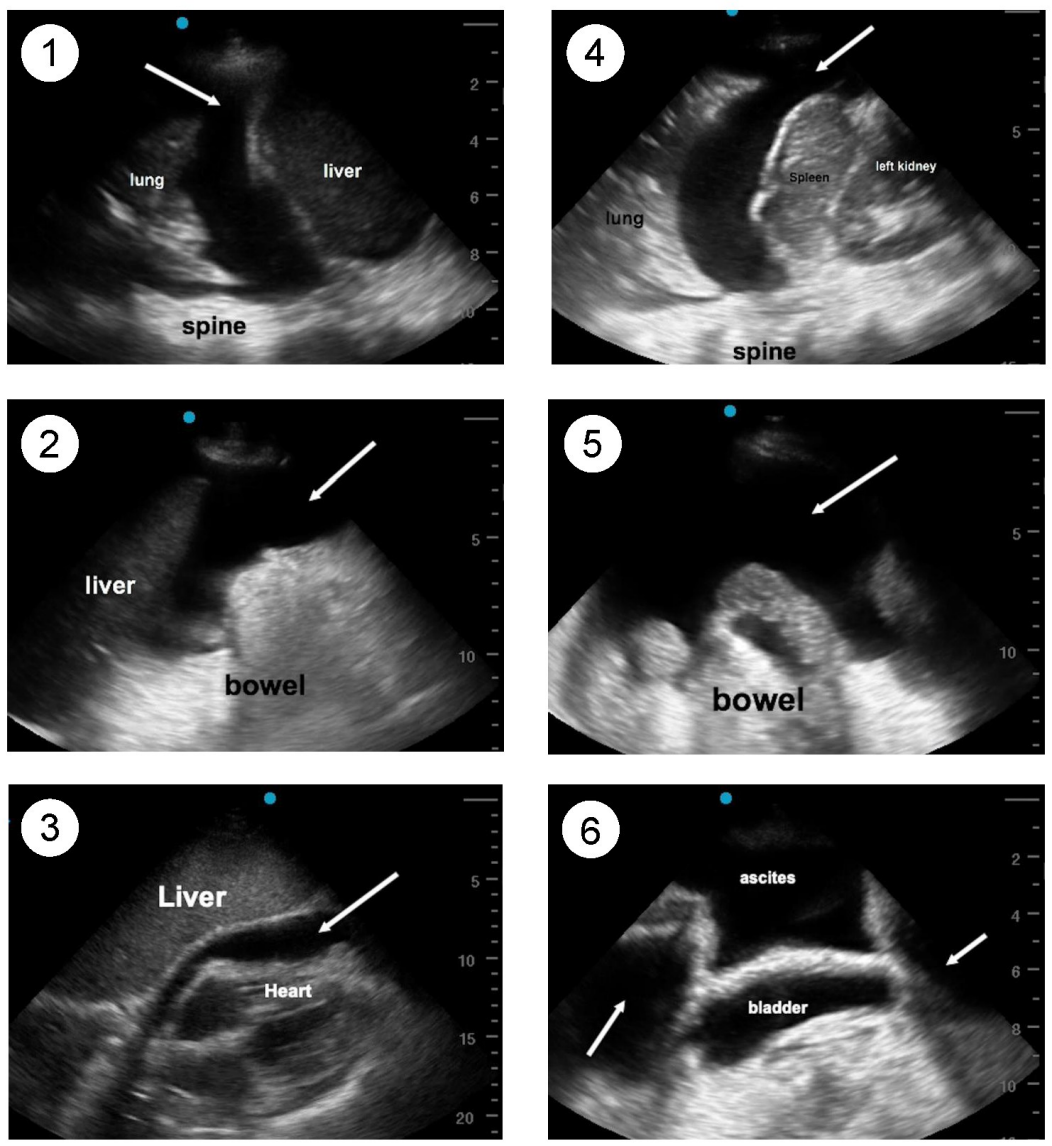
Sample FASC Examination images. FASC position 1: right pleural effusion. FASC position 2: right lower abdominal ascites. FASC position 3: a pericardial effusion. FASC position 4: a left pleural effusion. FASC position 5: lower abdominal quadrant ascites. FASC position 6: pelvic ascites around the bladder. White arrows indicate anechoic fluid collections.

The ultrasound probe should initially be placed in the mid-axillary line at the level of the xiphoid process with the indicator towards the patient’s head. The diaphragm should be visualized along with the liver in position 1 and the spleen in position 4 (Figure 1). In addition, the kidneys and thoracic spine should be identified in the abdomen. This usually requires sliding up and down between ribs spaces, sweeping the probe posteriorly, and tilting in the anterior-posterior plane. The thoracic spine can be identified because ultrasound waves reflect of bone, creating the hyperechoic appearance of the vertebral bodies with anechoic shadows deep to them. With the probe in the mid-axillary line, the vertebral column can be identified at the bottom of the screen. Normal aerated lung does not provide an acoustic window to visualize any deeper structures, thus the spine is only seen when there is pathology at the lung base, such as a pleural effusion. Visualization of the thoracic spine above the diaphragm is referred to as the “spine sign,” which confirms that pathology (such as pleural effusion) is not an artifact. The absence of a pleural effusion is often noted by a positive “lung curtain sign,” in which pleural sliding and A-lines are seen at the lung base during inspiration 12. When present, a pleural effusion is anechoic (black) and if large enough will lead to atelectasis of the nearby lung (Figure 2, Supplementary Video 1, 2). Loculations can be present in highly exudative pleural fluid. It is important to identify the thoracic spine posterior to pleural effusions (positive “spine sign”) to rule out artifact 13. Rib artifacts and reflection across the diaphragm can lead to hypoechoic and anechoic findings above the diaphragm, but these will not have a positive spine sign. 

In the supine position, ascites collects in the dependent locations, such as the sub-diaphragmatic space (Figure 2). Sliding the probe inferiorly from the diaphragm level when in position 1 and 4 allows for identification of the hepatorenal recess (Morrison’s pouch) and splenorenal recess, respectively. This fluid will be detected by its anechoic (black) appearance on ultrasound and requires correct identification of nearby structures. A fluid filled stomach, loops of bowel, or renal cysts can mimic ascites. 

### Position 2 and 5. Detecting Ascites in the lower abdominal quadrants

The probe is placed on the right lower or left lower abdominal quadrants and should be tilted anterior to posterior and rocked cranial to caudad to visualize positions 2 and 5 (Supplementary Video 3, 4). In abdominal carcinomatosis, ascites might be loculated with hyper-echoic (white) strands in the anechoic (black) fluid. 

### Position 3. Subxiphoid Cardiac Window to Detect Pericardial Effusion

Pericardial effusions are common in patients with cancer and can lead to cardiac tamponade, particularly if the fluid accumulates rapidly 14. The subxiphoid cardiac view is ideal for assessing the presence or absence of pericardial effusion (Figure 2, Supplementary Video 5). The probe should be placed inferior to the xiphoid process with the indicator pointed to the patient’s left (cardiology orientation). Care should be taken not to apply excessive pressure. The ultrasound probe is rocked to the patient’s left, using the liver as an acoustic window to view the heart and pericardial space. When present, a pericardial effusion is anechoic (black), but can have echogenic loculations or fibrin strands 11. Some pericardial effusions are mobile and collect in dependent areas based on positioning 11. Assessing for cardiac tamponade physiology is a more advanced POCUS skill: new, symptomatic, or large pericardial effusions in patients with cancer found on FASC examination should prompt referral for further evaluation. 

### Position 6. Pelvic view to detect ascites

The pelvis is another dependent area where ascites can be visualized. The probe is placed superior to the pubic symphysis and pointed inferiorly and posteriorly with the indicator to patient’s right (Supplementary Video 6). The probe can be tilted anteriorly to posteriorly and rocked left to right to improve visualization. It is important to identify the bladder so that urine is not mistaken for ascites. Ascites, when present, can be visualized anterior to, posterior to, or lateral to the bladder. 

## Conclusions

Pleural effusions, pericardial effusions, and ascites are commonly diagnosed in patients with cancer. POCUS is a useful adjunct to the physical examination in detecting, monitoring, and draining these effusions. While POCUS is not currently in widespread use by oncologists, trainees are increasingly learning POCUS in medical school and internal medicine residency 15, 16, 17, 18, 19, 20, 21. The FASC protocol contains views that are easily obtained and have been validated for use by clinicians in many other specialties. We anticipate that oncologists will find learning and independently performing the FASC protocol very rewarding. The FASC examination might allow oncologists to monitor their patients in clinic and in the hospital for fluid accumulation. For oncologists who are not trained in POCUS, there are many opportunities to learn POCUS in the continuing medical education setting. Emergency medical providers, hospitalists, and primary care providers currently using POCUS can easily incorporate the FASC examination into their practice 22, 23, 24. Further work is necessary to determine the right amount of training to competently perform the FASC examination.

## Conflicts of Interest

None declared.

## Supplementary Material

Video S1 Video clip of FASC position 1: right pleural effusion.

Video S2 Video clip of FASC position 4: left pleural effusion.

Video S3 Video clip of FASC position 2: right lower abdominal ascites.

Video S4 Video clip of FASC position 5: lower abdominal quadrant ascites.

Video S5 Video clip of FASC position 3: pericardial fluid.

Video S6 Video clip of FASC position 6: pelvic ascites around the bladder.
